# CSPP-L Associates with the Desmosome of Polarized Epithelial Cells and Is Required for Normal Spheroid Formation

**DOI:** 10.1371/journal.pone.0134789

**Published:** 2015-08-04

**Authors:** Johan Sternemalm, Stefan Geimer, Kari-Anne M. Frikstad, Kay O. Schink, Trond Stokke, Sebastian Patzke

**Affiliations:** 1 Department of Radiation Biology, Division of Cancer Medicine, Surgery and Transplantation, Institute for Cancer Research, Oslo University Hospitals–Norwegian Radium Hospital, Oslo, Norway; 2 Cell Biology/Electron Microscopy, University of Bayreuth, Bayreuth, Germany; 3 Department of Molecular Cell Biology, Division of Cancer Medicine, Surgery and Transplantation, Institute for Cancer Research, Oslo University Hospitals–Norwegian Radium Hospital, Oslo, Norway; 4 Centre for Cancer Biomedicine, Faculty of Medicine, University of Oslo, Oslo, Norway; RWTH Aachen, GERMANY

## Abstract

Deleterious mutations of the Centrosome/Spindle Pole associated Protein 1 gene, *CSPP1*, are causative for Joubert-syndrome and Joubert-related developmental disorders. These disorders are defined by a characteristic mal-development of the brain, but frequently involve renal and hepatic cyst formation. CSPP-L, the large protein isoform of *CSPP1* localizes to microtubule ends of the mitotic mid-spindle and the ciliary axoneme, and is required for ciliogenesis. We here report the microtubule independent but Desmoplakin dependent localization of CSPP-L to Desmosomes in apical-basal polarized epithelial cells. Importantly, siRNA conferred depletion of CSPP-L or Desmoplakin promoted multi-lumen spheroid formation in 3D-cultures of non-ciliated human colon carcinoma Caco-2 cells. Multi-lumen spheroids of *CSPP1* siRNA transfectants showed disrupted apical cell junction localization of the cytoskeleton organizing RhoGEF ECT2. Our results hence identify a novel, non-ciliary role for CSPP-L in epithelial morphogenesis.

## Introduction

Tissue morphogenesis and homeostasis are controlled by developmental signalling pathways, such as Hedgehog- and Wnt-pathways, which co-ordinate proliferation, differentiation, polarization and positioning of individual cells. These pathways regulate expression and activity of proteins that control remodelling of microtubules (MTs), actin- and intermediate filaments to shape cell morphology/stability and to form an intra-cellular scaffold for polarized transport of macro-molecules and vesicles. Filament orientation with respect to neighbouring cells is hence a critical factor for tissue morphogenesis. In epithelial tissues stable cell junctions are formed between neighbouring cells. They increase mechanical stability, promote junction based cell-cell communication, and are attachment sites and thus spatial reference points for cytoskeletal filaments at the cell cortex. Three types of junctions are distinguished within the junctional complex of apical-basal polarized epithelial cells: tight junctions (TJ), adherens junctions (AJ) and desmosomes. AJs and desmosomes provide strong intercellular cadherin based cell-cell adhesions, whereas TJs function in sealing the para-cellular space. AJs and desmosomes share a similar tripartite modular organization: Transmembrane, junction specific cadherin-family proteins form intercellular bridges and recruit at their intracellular tail armadillo-family proteins that provide docking sites for cytoskeleton linker proteins like β-catenin and Desmoplakin (for a review [1]). Interestingly, in epithelial cells grown in organoid 3D-culture the cytokinetic bridge determines the site for deposition of the apical membrane. Correct positioning of the cleavage furrow within the cell-cell context is therefore critical for symmetric growth and single lumen formation [2]. Furthermore, regulatory components of the cytokinetic apparatus itself are also involved in cytoskeletal organization at epithelial cell junctions [3]. Importantly, loss of cadherin based cell junction integrity interferes with differentiation, migration potential and polarization, and is associated with several pathologies, including cancer and inherited disorders [4–13].

The primary cilium is a compartmentalized membrane extrusion enriched for signal receptors. It is a pivotal organelle for several signalling pathways that initiate the transcriptional programs that prime cell-fate,-morphology and–function. These include in addition to the Hedgehog- and Wnt-signalling pathways mentioned above, also Notch-, PDGFRα, TGFβ, and Calcium signalling pathways (reviewed in [14]). The primary cilium is formed by a MT axoneme that is templated by the mother centriole of the centrosome, which is immobilized at the cell membrane. Due to its structure and function the primary cilium is considered as “a cellular antenna” for the extra-cellular cues (for a review [14]). De-regulation of cilia mediated signalling pathways has important implications for epithelial homeostasis and can promote malignant transformation and cancer progression [15–18]. Most importantly, a growing list of inherited human developmental disorders is correlated to structurally or functionally deficient primary cilia, and was hence collectively termed ciliopathies (for recent reviews [19,20]). Many of these ciliopathy causing genes interact genetically and encode for proteins engaged in cilia assembly, maintenance or ciliary signal transduction. Joubert syndrome and Joubert related syndrome(s) are examples of multi-organ ciliopathies [21]. Joubert syndrome is characterized by a unique malformation of the hindbrain (“molar tooth sign”) but patients most frequently present with extra-neurological findings including general developmental delay, retinal degeneration, skeletal malformation, and renal and hepatic epithelial cysts. Disruptive mutations in *Centrosome/Spindle Pole associated Protein 1* (*CSPP1*, *JBTS21*) were recently identified to be a major cause for Joubert syndrome [22–24]. The large protein isoform CSPP-L, encoded by *CSPP1*, is a ciliary/centrosomal protein required for cilia formation *in vitro* and *in vivo*. CSPP-L is localized to the (+)-ends of the axonemal MTs and the transition zone at the ciliary base [25]. Notably, CSPP-L is also a positive regulator of cytokinesis: CSPP-L localizes to the centrosome and kinetochore MTs of pro-metaphase/metaphase cells. CSPP-L translocates to the mid-spindle in late anaphase [26], where it is essential for the recruitment of the guanine nucleotide exchange factor and ECT2 binding protein MyoGEF to the central spindle and cleavage furrow [27]. ECT2 is also an important regulator of Rho activity downstream of E-cadherin based cell junctions, to which it is recruited in a centralspindlin complex dependent manner [3,28,29]. Several human ciliopathy proteins show extra-ciliary localization to cell junctions, but their function at this localization is not fully understood. Examples include PKD1, PKD2, NPHP1, NPHP4, SDCCAG8 and CEP164, which localize to cell junctions and are mutated in patients with inherited cystic kidney disease [30–35]. Previous studies on CSPP1 proteins were mostly limited to epithelial cell lines that do not form cell junctions. In the present study we therefore investigated the expression of CSPP-L in cell junction forming, apical-basal polarized HCC1937 breast cancer and Caco-2 colon cancer cells in 2D and 3D (organoid) cell culture. We report the localization of CSPP-L to apical cell junctions and describe a requirement for CSPP-L in normal spheroid formation.

## Materials and Methods

### Cell lines, cell culture and siRNA transfection

The breast cancer cell line HCC1937 (CRL-2336) and the human colon carcinoma cell line Caco-2 (HTB-37) were acquired from ATCC (Manassas, VA, US). HCC1937 cells were cultivated in RPMI1640 medium (Sigma-Aldrich, St.Louis, MO, US), supplemented with 10% fetal bovine serum (FBS) and antibiotics Penicillin and Streptomycin. Caco-2 cells were cultivated in DMEM (Life Technologies, Carlsbad, CA, US) supplemented with 15% FBS. For the calcium switch assay, cells were seeded in calcium free DMEM (Life Technologies) supplemented with 5% FBS and L-glutamine (denoted “low calcium medium”, due to low calcium concentration in FBS).

Cells were transfected with siRNAs in 6-well plates using Lipofectamin RNAiMAX (Life Technologies). Briefly, 100 pmol siRNA and 5μl RNAiMAX were diluted separately in 250 μl Optimem (37°C) (Life Technologies), mixtures combined and incubated for 20 min at room temperature to allow complex formation, supplemented with 500μl Optimem and added to cells. Six hours post-transfection 1 ml pre-warmed complete growth medium was added. *CSPP1* and *GFP* specific siRNAs were described earlier [25]. esiRNA targeting Desmoplakin were acquired from Sigma-Aldrich (EHU007001). For 3D-cultures cells were trypsinized 24hours post-transfection, re-suspended in 156 μl DMEM and mixed with 8 μl 1 M Hepes, 80 μl rat collagene (Sigma Aldrich) and 160 μl Matrigel (growth factor reduced, BD Biosciences, San Jose, CA, US). Cells were seeded in 8-well plates at 100μl/well (BD Biosciences) and the matrix was allowed to solidify for one hour before addition of 400 μl complete growth medium. 3D-cultures were analyzed after 5 days.

### Immunofluorescence staining and Imaging

For immunofluorescence imaging (IF) of HCC1937 monolayer cultures cells were grown on sterilized glass coverslips N1.5 (Glasswarenfabrik Karl Hecht KG, Sondheim, DE). Cells were fixed for 10 min in 100% ice cold methanol (-20C) and washed twice in phosphate-buffered saline (PBS) prior to blocking for 30 min in PBS containing 1% bovine serum albumin and 0.5% Triton X-100 (PBSAT). All antibody incubations were performed in PBSAT. Cells were incubated with primary antibodies for 2h at room temperature washed with PBS, and incubated with fluorescence labeled secondary antibodies for 45 min. Cells were counterstained for DNA using Hoechst 33258, washed in distilled water, air dryed and mounted on slides using Prolong Gold (Life Technologies). All images were obtained using appropriate optical filter settings on a multifluorescent sub-micron-bead calibrated (Life Technologies) AxioImager Z1 ApoTome microscope system (Carl Zeiss, Jena, Germany) equipped with Plan-Apochromat lenses (100x/NA1.40; 63x/NA1.40) and an AxioCam MRm camera. To display the entire cell volume, images are presented as maximal projections of z-stacks using Axiovision 4.8.2 (Carl Zeiss). Co-localization analysis was perfomed using the Co-localization analysis module in AxioVision 4.8.2 (Carl Zeiss).

Caco-2 spheroids were fixated in 10% neutral buffered formaline (Sigma-Aldrich) for 10 min at room temperature and then washed twice in PBS. Antibody incubation steps are the same as above. During the last step the media chamber is removed for slide analysis with the removal tool, air dryed and mounted under a No1.5 coverslip using Prolong Gold (Life technologies). Spheroids were imaged by phase-contrast on a CellObserver microscope system equipped with a 40x/NA1.3 Plan-Apochromat lense and an AcioCamMRm camera (Carl Zeiss). IF imaging of spheroids was performed on an AxioImager Z1 ApoTome microscope system described above. Optical sections were de-convolved using nearest neighbor algorithm in AxioVision Deconvolution module.

3D SIM imaging was performed using a Deltavision OMX V4 microscope (GE Healthcare, Little Chalfont, UK) equipped with three water-cooled PCO.edge sCMOS cameras, 405 nm, 488 nm, 568 nm and 642 nm laserlines and a 60x 1.42NA Plan-Apochromat lense (Olympus, Tokyo, JP). z-Stacks covering the whole cell, with sections spaced 0.125 mm apart, were recorded. For each z-section, 15 raw images (three rotations with five phases each) were acquired and the final super-resolution images were reconstructed using softWoRx software (GE Healthcare).

### Electron microscopy

Small pieces (about 2 mm^2^) of mouse trachea were fixed in MT-buffer (30 mM HEPES, 5 mM Na-EGTA, 15 mM KCl, pH 7.0) containing 3.5% formaldehyde for 2–3 hours at 4°C. After two brief washes with MT-buffer the tissue was dehydrated to 100% ethanol (30% and 50% ethanol on ice; then 70%, 95%, 100% ethanol at -20°C, 15 min each). Infiltration of the samples with LR Gold resin (London Resin Company, Reading, GB) was performed at -20°C according to the following scheme: LR Gold/ethanol (1:3) for 2 h, LR Gold/ethanol (3:1) for 4 h, LR Gold containing 0.4% benzil for 36 h (with several changes of the medium). Polymerization was performed under fluorescent light for 48 h at -20°C. Ultrathin sections (60–80 nm) were cut with a diamond knive (type ultra 35°; Diatome, Biel, CH) on a EM UC6 ultramicrotome (Leica Microsystems, Wetzlar, DE) and mounted on pioloform-coated, single-slot gilded copper grids (Science Services, Munich, DE). For immunolabeling, the sections were blocked for 1–2 hrs at room temperature with blocking buffer (2% BSA, 0.1% fish gelatin and 0.05% Tween 20 in PBS; pH 7.4) and incubated in anti-CSPPL antibody (polyclonal, rabbit, diluted 1:200 in blocking buffer) overnight at 4°C. Grids were washed 3–5 times with PBS containing 0.15% BSA-c (Aurion, Wageningen, NL) for 10 min each and incubated for 1.5 hrs with 15-nm gold particles conjugated to goat anti-rabbit IgGs (British Biocell, Cardiff, GB) diluted 1:30 in blocking buffer. Grids were washed 3–5 times with PBS containing 0.15% BSA-c for 10 min each, fixed for 8 min in 1% glutaraldehyde in PBS and washed 3 times for 5 min each in distilled water. After immunolabeling, the sections were stained with uranyl acetate and lead citrate [36] and viewed with a JEM-2100 transmission electron microscope (JEOL, Tokyo, JP) operated at 80 kV. Micrographs were taken using a 4,080 x 4,080 pixels charge-coupled device camera (UltraScan 4000, Gatan, Pleasanton, CA, US) and Gatan Digital Micrograph software (version 1.70.16). Image brightness and contrast were adjusted using Adobe Photoshop 8.0.1.

### Antibodies and fluorescent labeled phalloidin

The following primary antibodies were used a-CSPP-L (Proteintech Europe, Manchester, UK), a-Desmoplakin (Millipore), a-β-catenin (BD Bioscience), a-α-tubulin (Sigma-Aldrich), a-E-cadherin (Cell signaling, Danvers, MA, US), a-PKCζ (Santa Cruz Biotechnology, Dallas, TX, US), a-Pericentrin (Abcam), a-acetyl.tubulin (Sigma-Aldrich), a-γ-tubulin (Sigma-Aldrich) and a-ECT2 (Santa Cruz Biotechnology). Atto647N labeled phalloidin was obtained from Sigma-Aldrich. Fluorochrome or HRP conjugated secondary antibodies were: donkey anti-rabbit and donkey anti-mouse (Jackson Immuno Research, West Grove, PA)

### Immunoblotting

Cells were lysed directly in 2x SDS sample loading buffer containing DTT and supplemented with Benzonase (Merck, Whitehouse Station, NJ, US) and Aproteinin (Sigma-Aldrich). Lysates were incubated for 5 min on ice to allow DNA degradation prior to denaturation for 7 minutes at 95C. Proteins were resolved by SDS polyacrylamide gel electrophoresis and transferred onto polyvinylidene difluoride membrane (Millipore, Billerica, MA). Western blot analysis and detection was described earlier [25].

## Results

### CSPP-L localizes to the Desmosome

**Fig 1:**
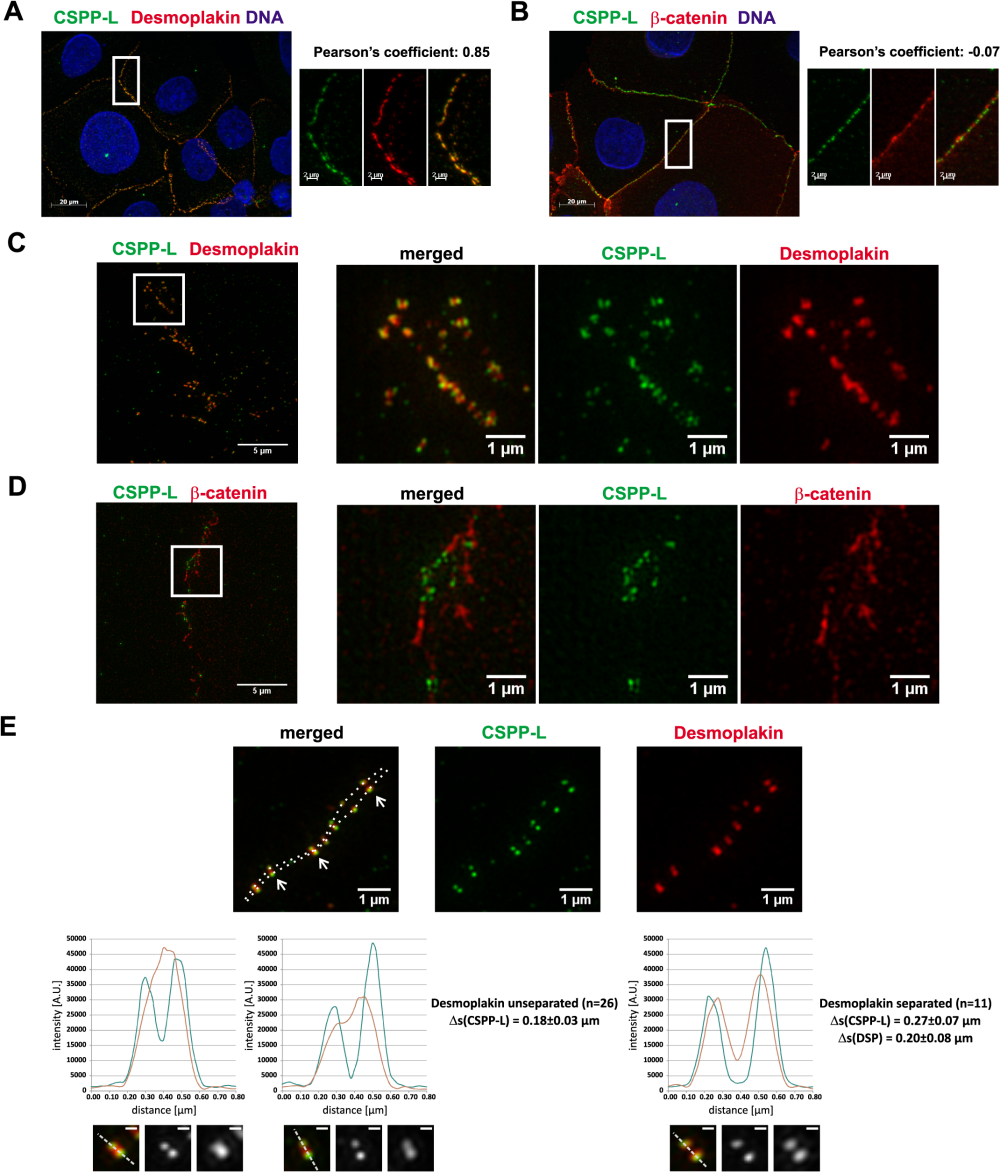
CSPP-L localizes to the Desmosome. IF of CSPP-L (a-CSPP-L, green) in the basal-like breast cancer cell line HCC1937 showed co-localization with the desmosomal protein Desmoplakin (A, a-DSP, red; upper panel) but not with the AJ associated protein β-catenin (B, a-CTNNB1, red; lower panel), as tested by co-localization analysis (linear dependence, Pearson’s coefficient). (C-E) 3D-super-resolution microscopy refined the localization of CSPP-L to single patches that frame Desmoplakin at the cell junction (C) and do not co-localize with β-catenin (D). Higher magnification image and line plots of signal intensities across individual desmosomes at cell junctions (E). Dashed line in overview image connects peaks in Desomplakin signals. Line plots show signal intensities along an 800nm line across desmosomes depicted below (dashed line in magnifications, scale bars magnifications = 200nm). Average distances (with standard deviation) of peak intensities of CSPP-L patches and Desmoplakin (26 cells with non-separated and eleven cells with separated Desmoplakin signals).

We recently reported the localization of CSPP-L at motile cilia of mouse trachea epithelial cells by immunogold and immunofluorescence analysis (IF) [37]. During the investigation of murine epithelial cells CSPP-L labelling at desmosomal junctions was noticed (S1 Fig). The staining of CSPP-L at undetermined apical cell junctions was seen earlier in apical-basal polarized Madin-Darbey canine kidney cells (MDCK, S1 Fig), but yet not characterized in human cell line models. We therefore examined the localization of CSPP-L in confluent, apical-basal polarized cells of the human basal-like breast cancer cell line HCC1937 (Fig 1). In congruence with the staining pattern of CSPP-L in MDCK cells, CSPP-L localized to apical cell junctions barely overlapping with the AJ protein β-catenin (Fig 1A; Pearson’s co-localization coefficient = -0.07) but co-localizing with the desmosomal protein Desmoplakin (Fig 1B; Pearson’s colocalization coefficient = 0.85) as determined by structured illumination microscopy (SIM, ApoTome [38]). The Desmosome is a highly organized, electron dense structure of less than 1 μm in diameter, which bridges cytoplasmic plaques of opposing cells via membrane transpassing desmosomal cadherins that bind within an approximately 35nm wide intercellular space ([4] and S1 Fig). Desmoplakin connects the membrane adjacent outer dense plaque with the intermediate filament organizing inner dense plaque. We applied three-dimensional super-resolution microscopy (3D-SIM, [39,40]) to resolve the localization of CSPP-L in HCC1937 cells at a lateral resolution of about 125nm (Fig 1C–1E). 3D-SIM revealed that CSPP-L is localized in paired patches that frame Desmoplakin labelled plaques at the cytolasmic site (Fig 1C). These paired patches are well separated from membrane associated β-catenin (Fig 1D). In most desmosomes the desmoplakin label of individual plaques was below the resolution limit (Fig 1E), while Desmoplakin framing CSPP-L patches were separated by 180±30nm. Interestingly, CSPP-L associated with the cytoplasmic side of desmosmal plaques prior to merging of Desmoplakin signals (Fig 1E).

From these results we concluded that CSPP-L is associated with the cytoplasmic side of desmosomal plaques protein in apical-basal polarized epithelial cells.

### CSPP-L is recruited to the desmosome in a Desmoplakin dependent and microtubule independent manner

**Fig 2:**
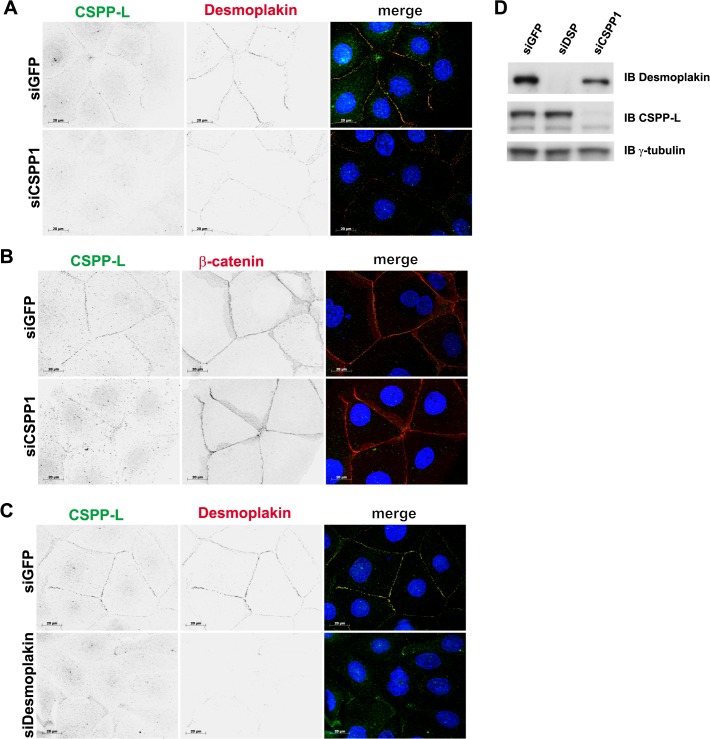
Desmoplakin is required for CSPP-L localization to the Desmosome. IF (A-C) and immunoblotting of total cell lysates (D) of HCC1937 cells treated with indicated siRNAs. Cells were transfected in low calcium medium. 72hrs post-transfection cells were allowed to form cell-cell junctions for 40 min by change to pre-warmed, normal calcium medium. Cell-cell junction staining of CSPP-L (green in overlay image) is siCSPP1 sensitive (A). Depletion of CSPP-L does not impair cell-cell junction localization of Desmoplakin (A, red) or β-catenin (B, red). Depletion of Desmoplakin (C, red) results in loss of CSPP-L (green) staining at cell-cell junctions. Knockdown efficacy was monitored by immunoblotting for Desmoplakin, CSPP-L and compared to the loading control γ-tubulin (D).

We next investigated if CSPP-L and Desmoplakin are interdependent for their sub-cellular localization to cell junctions. Initial experiments using transfection of confluent HCC1937 cells with siRNAs targeting *CSPP1* or *DSP* mRNAs did not result in any marked decrease in cell-cell contact staining (data not shown). We hence speculated that siRNA mediated depletion of these proteins at desmosomes might be impaired by slow turn-over of CSPP-L within cell-cell contact protein complexes. Calcium is an essential co-factor for cadherin based cell-cell contact formation, which is inhibited at low calcium concentration and inducible by restoring normal calcium levels through re-addition of normal growth medium (calcium-switch). We therefore transfected HCC1937 cells at sub-confluence with *CSPP1* or *DSP* targeting siRNAs in medium containing low calcium concentration. 72 hours post siRNA transfection calcium levels were restored and cell-cell contacts allowed to form for 40 minutes. Cell-cell contact formation was monitored by IF (Fig 2A–2C) and knock-down efficacy also analyzed by immunoblotting for Desmoplakin and CSPP-L in total cell lysates (Fig 2D). GFP targeting control siRNA transfectants readily formed CSPP-L and Desmoplakin comprising patches at cell junctions. siRNA mediated knock-down of CSPP-L slightly decreased, but did not abolish Desmoplakin staining at cell-cell contacts (Fig 2A). β-catenin staining at cell junctions in siCSPP1 transfectants was indistinguishable from siGFP control transfected HCC1937 cells (Fig 2B). In contrast, siRNA mediated depletion of Desmoplakin strongly decreased CSPP-L staining at cell junctions (Fig 2C).

**Fig 3:**
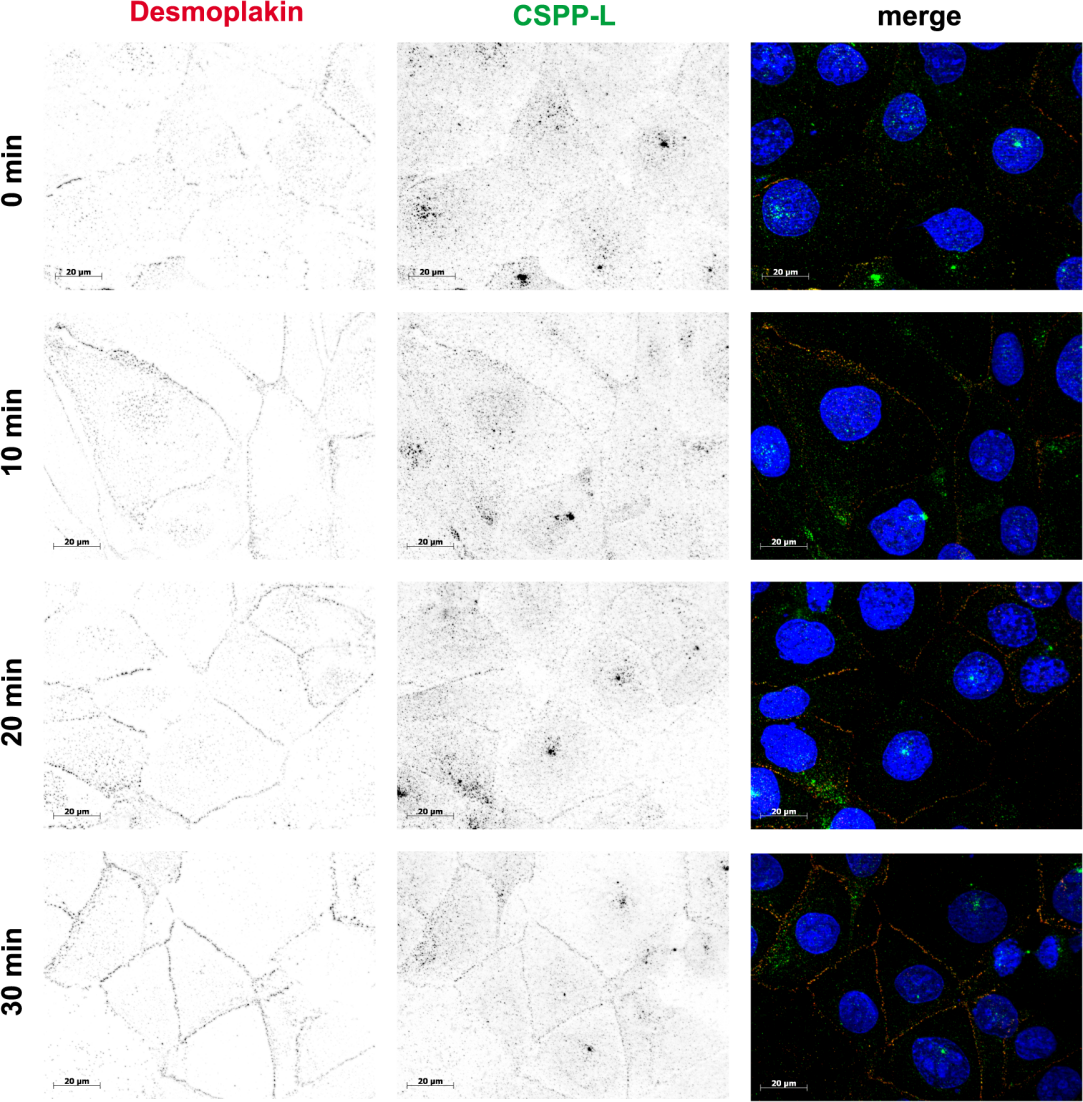
Desomoplakin precedes CSPP-L localization to forming cell-cell junctions. IF of Desmoplakin (red) and CSPP-L (green) localization during calcium switch induced cell junction formation in HCC1937 cells. Cells were grown to confluency at low calcium concentration. Cells were analyzed at indicated time points after change to normal calcium medium. Desmoplakin preceded CSPP-L localization to new formed cell-cell junctions.

**Fig 4:**
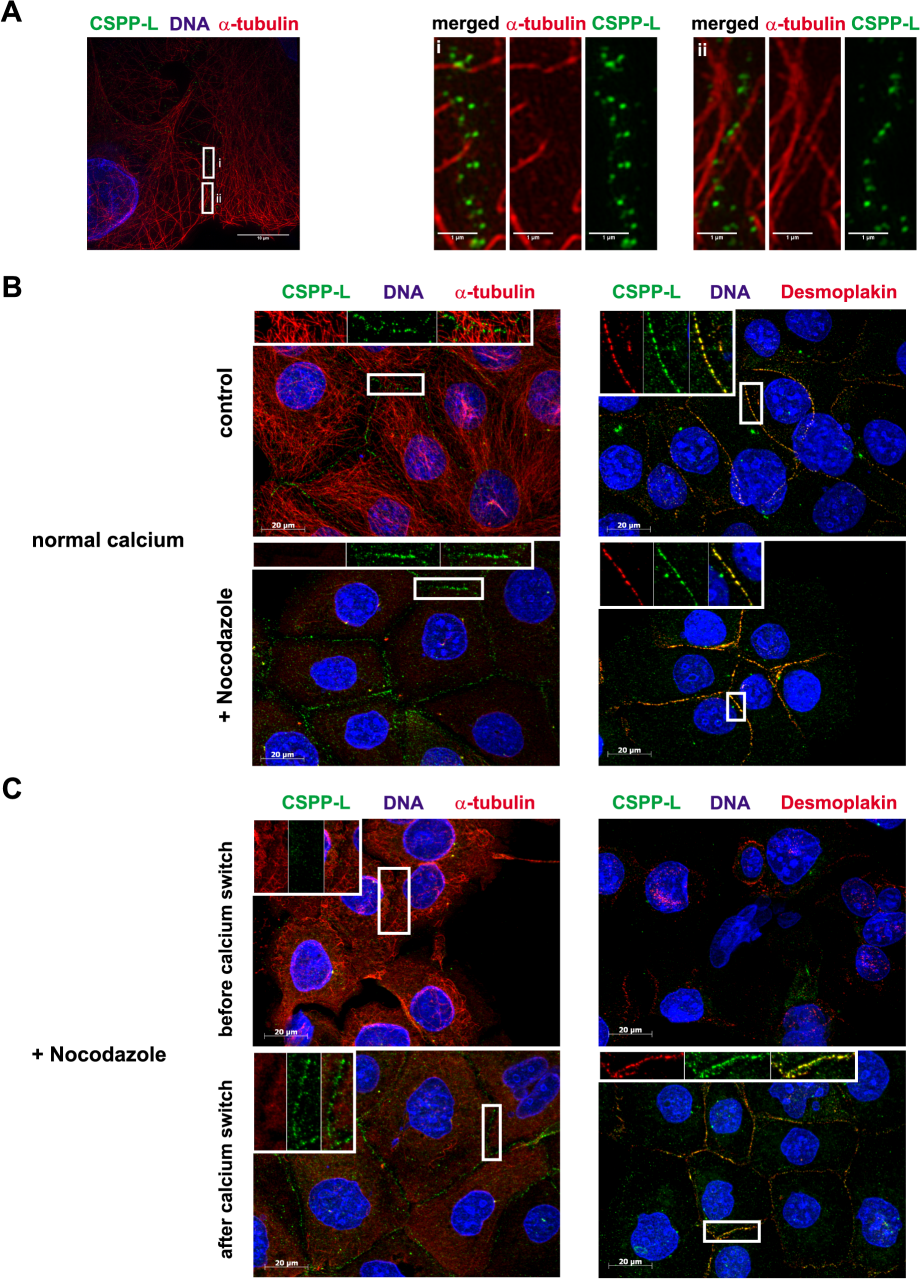
Microtubule independent localization of Desomoplakin and CSPP-L to the Desmosome. (A) 3D-super-resolution microscopy of CSPP-L (green) and MTs (α-tubulin, red). MTs are infrequently observed to localize with MT (+)-ends at junctional CSPP-L pairs (i), but predominantly align parallel to the cell cortex (ii). (B) Cell junction staining of CSPP-L (green) and Desmoplakin (red, right panel) in HCC1937 cells is resistant to nocodazole induced MT de-polymerization (α-tubulin, red, left panel). (C) MTs are not required for recruitment of Desmoplakin and CSPP-L to forming cell junctions in a calcium switch assay in HCC1937 cells.

We concluded from these results that cell-cell contact staining of CSPP-L is strictly dependent on expression of Desmoplakin. CSPP-L partially contributes to stabilization of Desmoplakin at cell junctions but is not required for recruitment of β-catenin to cell junctions. We therefore investigated the timing of Desmoplakin and CSPP-L recruitment to cell-cell contacts in HCC1937 cells in response to switch from low to normal calcium levels (Fig 3). Under low calcium conditions (0 min) some Desmoplakin localized in discontinuous patches along cell-cell contacts, whilst CSPP-L was almost exclusively absent from cell-cell contacts. Ten minutes after reconstitution of normal calcium levels Desmoplakin but not CSPP-L decorated all cell-cell contacts. Complete co-occurrence of Desmoplakin and CSPP-L at cell-cell contacts was not observed until 30 min after calcium reconstitution. These results suggested that during desmosomal plaque assembly in calcium-switch experiments Desmoplakin precedes CSPP-L at the forming cell junction. Since CSPP-L localizes to MT (+)-ends of cilia axonemes and the central spindle apparatus, we tested if CSPP-L localization to the desmosomal plaque may rely on an intact MT cytoskeleton (Fig 4). CSPP-L was not observed on MT (+)-ends within the cytoplasm (Fig 4A and data not shown). However, infrequently MT (+)-ends were observed to localize head-on at junctional CSPP-L pairs in 3D-SIM (Fig 4A i). The majority of MTs aligned parallel to the cell cortex (Fig 4A ii). This result indicated that CSPP-L and MT-ends may at least temporarily coincide at the Desmosome. To test if MTs are required for maintenance of CSPP-L at the desmosome, HCC1937 cells were grown to confluency to allow desmosomal plaque formation and then treated with the MT polymerization inhibiting drug Nocodazole (30 μM f.c. / 30 min) (Fig 4B). De-polymerization of the MT cytoskeleton did not alter the cell junction localization of Desmoplakin or CSPP-L. We next examined if MTs are required for recruitment of CSPP-L to the desmosome in a calcium-switch experiment (Fig 4C). HCC1937 were grown in low calcium media and exposed to Nocodazole for 30 min prior to and after reconstitution of physiological calcium concentration. Desmoplakin and CSPP-L were also under these conditions efficiently recruited to cell junctions. We conclude from these results that CSPP-L and Desmoplakin assemble and maintain at cell junctions in a MT independent manner, but may interact with MT (+)-ends at cell junctions.

### CSPP-L depletion impairs spheroid morphogenesis of Caco-2 cells in 3D-culture

**Fig 5:**
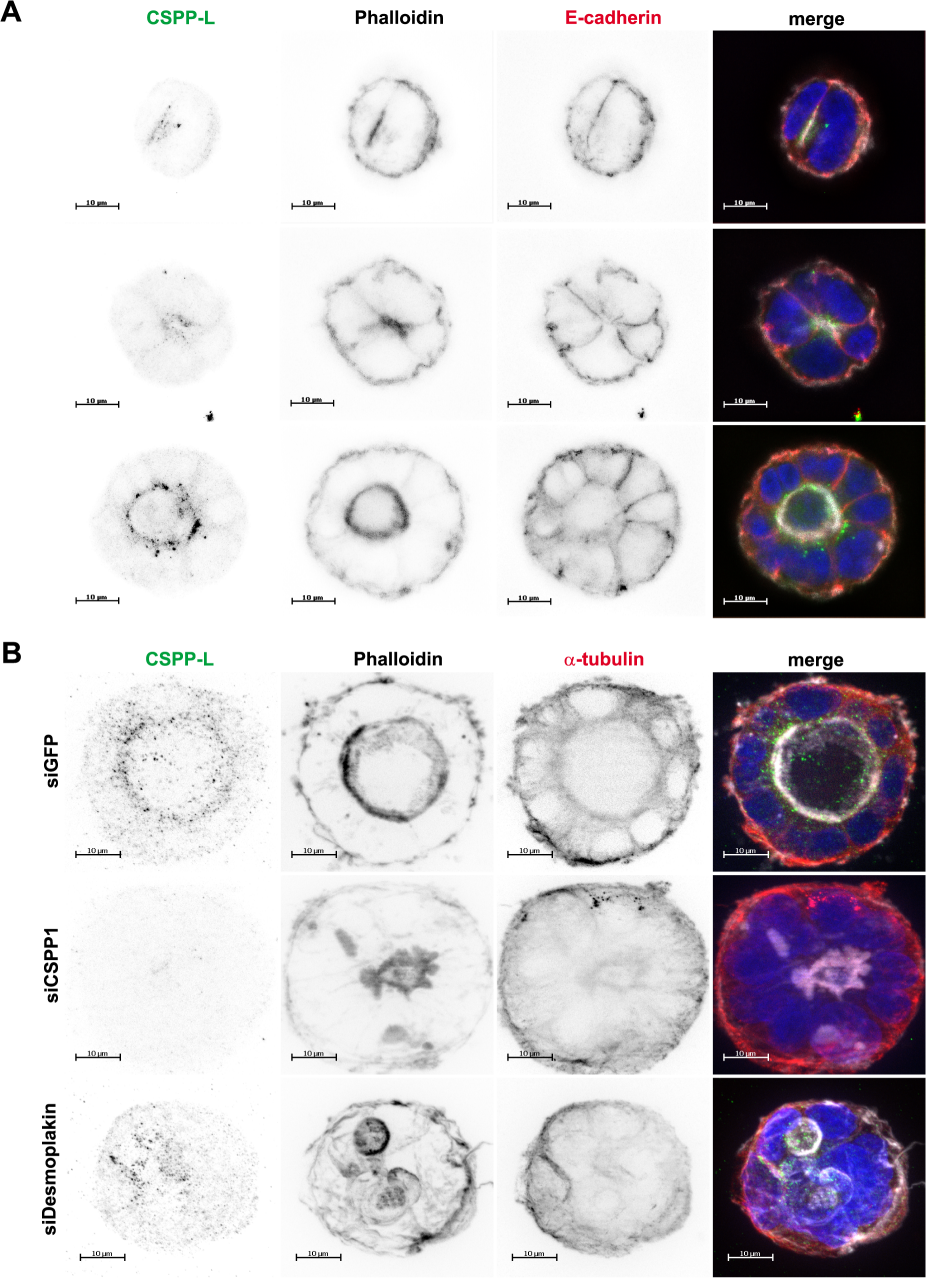
Depletion of CSPP-L and Desmoplakin cause multi-lumen spheroid formation in Caco-2 spheroids. (A) Localization of CSPP-L (a-CSPP-L, green), filamentous actin (Phalloidin, white), E-cadherin (a-E-cadherin, red), and DNA (blue) during different stages of spheroid development of Caco-2 cells. Cells were grown in 3D-Matrigel culture and formalin fixed for IF. Images show projections of z-sections enclosing the entire lumen volume. CSPP-L shows prominent enrichment juxtapose to the apical filamentous actin throughout all stages of spheroid development (B) The apical CSPP-L staining pattern (a-CSPP-L, green) is CSPP1 siRNA sensitive and not altered by Desmoplakin depletion (a-α-tubulin, red; phalloidin, white). CSPP1 and Desmoplakin siRNA Caco-2 transfectants develop disorganized cell aggregates with multiple lumen.

**Fig 6:**
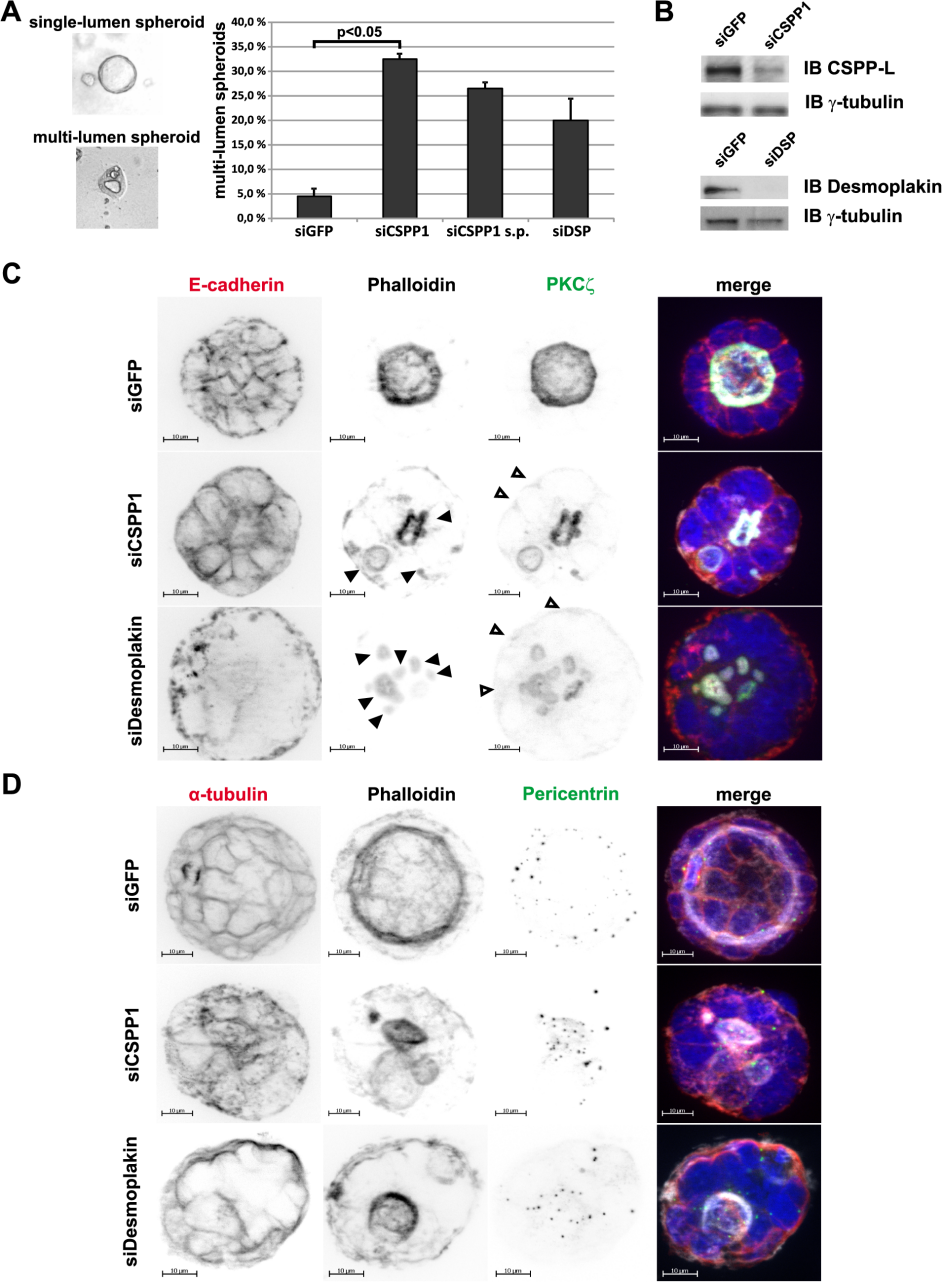
Apical-basal polarity is not disrupted in CSPP-L and Desmoplakin depleted multi-lumen Caco-2 spheroids. (A) Quantification of the multi-lumen phenotype in Caco-2 siRNA transfectants (siGFP, siCSPP1, siCSPP1 *s*mart *p*ool, siDSP). The bar diagram shows average of two experiments, error bars depict standard deviation. Statistical significance was tested by paired t-test. (B) CSPP-L and Desmoplakin depletion in Caco-2 spheroids was validated by immunoblotting (right panel). (C) Multi-lumen spheroids in CSPP1 and Desmoplakin depleted cells depict filamentous actin stabilization indicated by solid arrow heads (Phalloidin, white) and PKCζ enrichment (a-PKCζ, red) at the lumen facing apical membrane. Occasional weak PKCζ staining at the basal-side of outer-rim cells is seen in *siCSPP1* and *siDSP* transfectants (open arrowheads). (D) Centrosomes (a-Pericentrin, green) positioned in the lumen oriented cytoplasm (see also S1, S2, and S3 Videos).

Desmosomal organization of intermediate filaments and columnar MTs integrity is an important factor in epithelial tissue morphogenesis and homeostasis. We therefore investigated the expression of CSPP-L in apical-basal polarized layers or spheres of intestinal epithelial Caco-2 cells and studied the effects of CSPP-L depletion on spheroid formation. CSPP-L and Desmoplakin co-localized at apical cell junctions of Caco-2 cell layers (S2 Fig), similar to the localization pattern in HCC1937 cells (Fig 1). To study the localization of CSPP-L in spheroids Caco-2 cells were seeded in a Matrigel-matrix, which promotes apical-basal polarization, spheroid growth and lumen formation [2]. IF of cells in Matrigel-matrix requires para-formaldehyde fixation for preservation of spheroid morphology. Unfortunately, para-formaldehyde fixation abrogated staining of CSPP-L and Desmoplakin at the desmosome in Caco-2 cell spheroids and monolayers (data not shown) and could hence not be evaluated at this compartment. However, at two-cell stage detectable CSPP-L prominently localized in a spotted pattern proximal to the central filamentous actin layer at the site of forming apical membrane and apical end of E-cadherin staining (Fig 5A). This localization pattern along the apical filamentous actin layer was observed throughout all stages of spheroid formation. The specificity of the cytoplasmic CSPP-L staining pattern was validated by transfection with *CSPP1* targeting siRNA (Fig 5B and Fig 6). Interestingly, CSPP-L depleted Caco-2 spheroids developed multiple lumen or multiple central filamentous actin structures. Furthermore, multi-lumen spheroids formed by Desmoplakin or CSPP-L depleted Caco-2 cells showed aberrant MT networks and depicted similar morphology (Fig 5B and Fig 6).

CSPP1 siRNA transfection led to a six-fold and Desmoplakin depletion to a five-fold increase in multi-lumen spheroid formation, when compared to GFP siRNA control transfectants (Fig 6A). Knockdown efficacy of CSPP-L and Desmoplakin was monitored by immunoblotting (Fig 6B). Apical and lateral membrane identity was largely unaltered in CSPP-L and Desmoplakin depletion induced multi-lumen spheroids as determined by staining for thel apical membrane protein kinase C-zeta (PKCζ) and lateral/basal membrane marker protein E-Cadherin (Fig 6C). Outer-rim cells with weak PKCζ staining at the basal-side were about three-fold increased in *CSPP1* and *Desmoplakin* siRNA transfectants, respectively, compared to siGFP transfectants (siCSPP1 = 17.4±2.6%, siDSP = 15.5±0.8%, siGFP = 6.4±2.3%; p<0.05 paired t-test, 50 spheroids per treatment in four experiments). However, occurrence of mislocalized PKCζ staining was limited to only few cells (1–3) of the outer-rim, always of low intensity and exclusively co-occuring with filamentous actin. Further, CSPP-L and Desmoplakin depletion did not affect the positioning of centrosomes (Fig 6D and S1, S2, S3 Videos) in the cytoplasmic space between nucleus and apical membrane (Phalloidin labelled filamentous actin). These results suggested that neither CSPP-L nor Desmoplakin are strictly required for establishing apical-basal polarity in Caco-2 cells.

**Fig 7:**
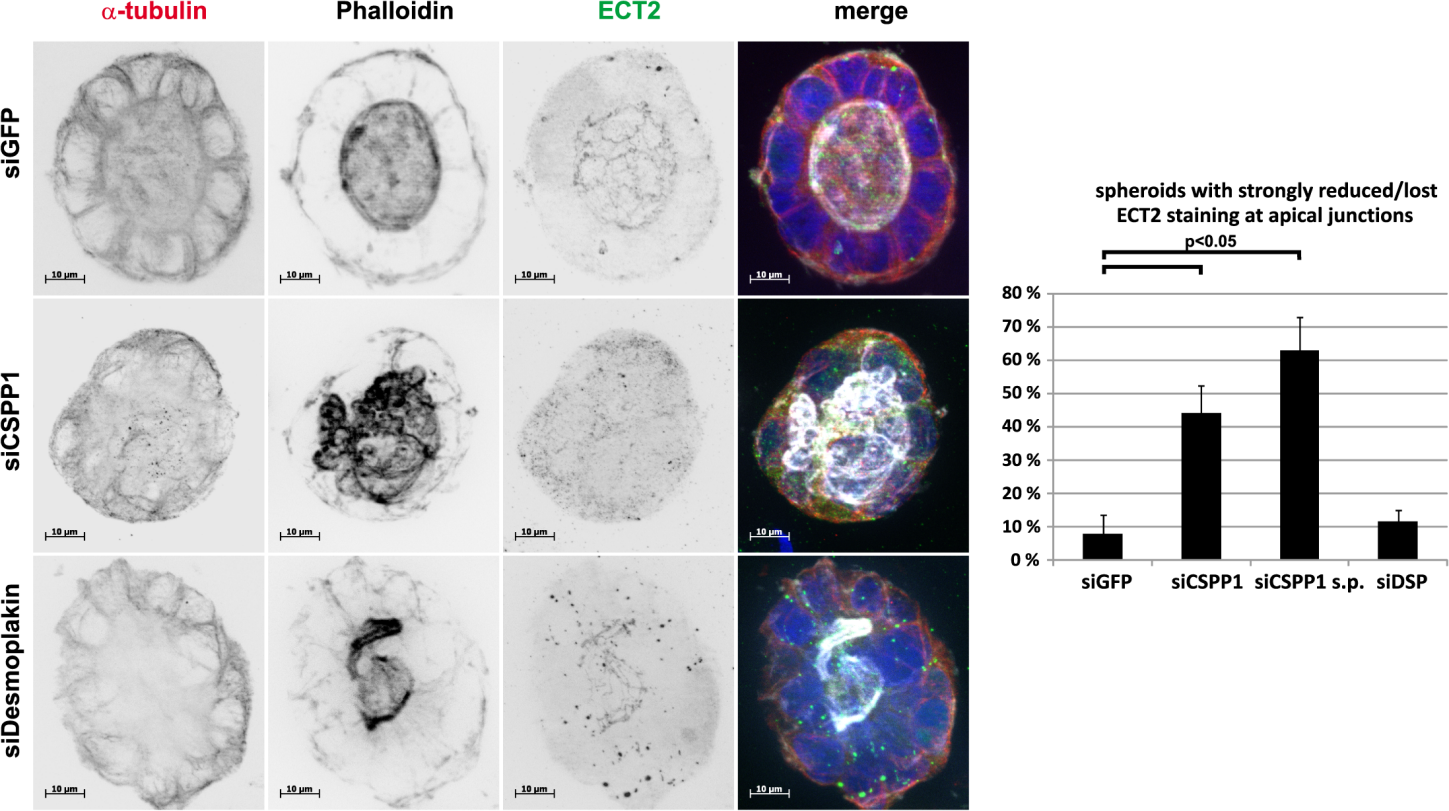
Mis-localization of ECT2 in CSPP1 depleted multi-lumen Caco-2 spheroids. Spheroids of Caco-2 cells transfected with indicated siRNAs were stained for ECT2 (a-ECT2, green), the filamentous actin (Phalloidin, white) and α-tubulin (a-α-tubulin,red). ECT2 localized to apical cell-cell junctions in single-lumen spheroids of siGFP control transfectants and mal-organized spheroids of Desmoplakin depleted cells. The apical cell-cell junction ECT2 staining pattern is lost in multi-lumen spheroids of CSPP-L depleted cells. Bar diagram shows frequency of spheroids with lost or strongly reduced junctional ECT2 staining (100 spheroids per treatment scored in two experiments, error bars depict SEM, statistical significance was tested in paired t-test).

The epithelial zonula adherens is an anchoring point for cortical MTs and weakening of junctional MTs can cause mechanic instability of epithelial cell layers [41–45]. The centralspindlin complex controls Rho activity and thereby the co-ordination of MT and actin dynamics at the cytokinetic furrow. The centralspindlin complex also controls Rho activity at the epithelial zonula adherens via recruitment of the Rho GEF ECT2 centralspindlin [3]. It was thus suggested that the zonula adherens assembles the interphase equivalent of the cytokinetic furrow. CSPP-L localizes to the mid-spindle during telophase and cytokinesis and is required for recruitment of the Myosin-II interacting-guanine nucleotide-exchange factor (MyoGEF) to the mid-spindle, which in turn is required for the recruitment of ECT2 to the cleavage furrow [27]. Interestingly, we found ECT2 to be misplaced from apical cell-junction in CSPP-L depleted multi-lumen Caco-2 spheroids, but not siGFP control transfectants (Fig 7) Notably, Desmoplakin depleted cells also differed from control cells in their ECT2 staining pattern, but retained ECT2 at cell junctions. Hence, CSPP-L dependent organization of ECT2 at apical junctions is Desmoplakin independent and may contribute to multi-lumen formation in Caco-2 spheroids.

## Discussion

Earlier studies in sub-confluent cell cultures of human epithelial cell lines identified CSPP-L as a centrosomal protein with a cell cycle phase dependent extra-centrosomal localization to MT (+)-ends of the ciliary axoneme in resting/terminally differentiated cells and the MT (+)-ends of the mitotic mid-spindle in dividing cells [25,26,46,47]. Our present investigation of monolayer or 3D-cultures of cell junction forming epithelial cells is the first to report a localization of CSPP-L to apical cell junctions. IF of CSPP-L and Desmoplakin in monolayers of human HCC1937 and Caco-2 as well as canine MDCK epithelial cells identified CSPP-L at the Desmosome (Fig 1, S1 Fig, S2 Fig). We show by super-resolution microscopy that CSPP-L uniformly localizes in single patches at the cytoplasmic side of Desmoplakin. The desmosomal localization of CSPP-L is Desmoplakin dependent (Fig 2) and occurred subsequently to Desmoplakin in Calcium-switch experiments (Fig 3). These results suggest that CSPP-L is not required for desmosome assembly. Further, decreased cell junction staining of CSPP-L was most prominent if CSPP-L was depleted prior to calcium induced cell junction formation (Fig 2 and data not shown), indicative for a low turn-over of CSPP-L at the formed desmosomal plaque. This interpretation is further supported by the nocodazole-resistant co-localization of CSPP-L and Desmoplakin at cell junctions (Fig 4). The exact function of CSPP-L at the desmosome remains to be elucidated. However, our data add CSPP-L to a list of centrosomal proteins with MT organizing/anchoring function that localize to the Desmosome. Ninein, Lis1, and Ndel are MT associated/anchoring proteins that are recruited from the centrosome to the desmosome in a Desmoplakin dependent manner in apical-basal polarized cells to control cortical MT organization [43–45,48–50]. Dynein/dynactin complex binding proteins CLIP170 and EB1 are, in addition to Lis1, further examples of MT end-binding proteins interacting with desmosomal junctions [44,45,51,52]. We showed earlier that CSPP-L localizes to MT (+)-ends of the mitotic mid-spindle and the ciliary axoneme [25,26]. Further, ectopically expressed CSPP-L associates with and over-stabilizes MTs [26]. It is therefore tempting to speculate that CSPP-L functions as linker protein in the spatio-temporal controlled stabilization of MTs, stabilizing MT (+)-ends at the centrosome, mid-spindle, ciliary axoneme and the desmosome. Supportively for this model, multi-lumen spheroids of CSPP-L depleted cells showed aberrant MT organization, but unaltered enrichment of filamentous actin and PKCζ at apical membranes (Figs 5–7). Impaired MT-cortex interaction in CSPP-L depleted cells could contribute to multi-lumen spheroid formation by decreasing mechanical stability of cell-cell layers, similar to the effects of Lis1 or Desmoplakin ablation [44]. MT-cortex interactions are also crucial for spindle orientation. Mal-orientation of the cell division plane in CDC42 depleted cells promotes amorphous/unequal growth and inappropriate deposition of apical membrane within the forming spheroid without affecting establishment of apical-basal polarity [2], phenotypically similar to the effect of CSPP-L depletion. Interestingly, CSPP-L is required for recruiting the Myosin-II interacting-guanine nucleotide-exchange factor (MyoGEF) MyoGEF and RhoGEF ECT2 to the mid-spindle of dividing HeLa cells [27]. Sub-cellular localization analysis in Caco-2 spheroids was limited by formalin fixation, which prevented staining of Desmoplakin and CSPP-L at cell junctions, but did not abrogate its cytoplasmic staining. We found that CSPP-L accumulated in a punctuate staining pattern at the apical region of Caco-2 cells throughout different stages of cyst formation (Fig 5) and that depletion of CSPP-L, but not Desmoplakin, is correlated with loss of ECT2 staining at apical cell-cell junctions (Fig 7). It is thus possible that CSPP-L is independently of Desmoplakin involved in deposition and/or retention of ECT2 at the zonula adherens after completion of cell division, putatively acting co-operatively with the centralspindlin complex [3]. Further work, including ultrastructural analysis of apical junctions in CSPP-L depleted cells, is required to corroborate this model.

Finally, mutations in *CSPP1* are a major cause of Joubert-syndrome and Joubert-related disease—ciliopathies in which affected individuals frequently present with renal and hepatic cysts. Our results may suggest that cyst formation in *CSPP1* could at least partially be attributed to a non-ciliary function of CSPP-L. Interestingly, a role in apical junction formation is suggested for ciliopathy proteins of the NPHP8-NPHP4-NPHP1 module [53], of which NPHP8 and NPHP4 can form a tripartite complex with CSPP-L [25]. Individual depletion of these ciliopathy proteins resulted in irregular lumen formation in IMCD3 3D spheroids, while ciliation was largely unaffected [53]. Moreover, two further JBTS-proteins, CEP164 and SDCCAG8, co-localize with the desmosome associated protein Ninein at cell-cell junctions of renal epithelial cells [35]. Their putative genetic or functional interaction with CSPP-L and the desmosomal junction should thus be investigated.

To conclude: we report the desmoplakin dependent localization of CSPP-L to apical cell junctions and identify a role for CSPP-L in spheroid formation of apical-basal polarized, non-ciliated epithelial cells.

## Supporting Information

S1 Fig CSPP-L localization to apical cell-cell junctions in mouse trachea epithelia cells and MDCK2 cell monolayers. (A) IF of apical-basal polarized MDCK2 cell-monolayers showing localization of CSPP-L (a-CSPP-L, green) to (A) centrosomes (a-γ-tubulin, red), (B) primary cilia (a-acetylated tubulin, red), and (C) apical cell-cell junctions (a-β-catenin, red). (D) Post-embedding immunogold labeling of CSPP-L in mouse trachea epithelia cells shows CSPP-L staining in the vicinity of the desmosomal junction. A proportionally scaled 3D-SIM image of a HCC1937 cell Desmosome is shown for comparison.
(TIF)

S2 Fig CSPP-L localization to apical cell-cell junctions in Caco-2 cell monolayers. IF of apical-basal polarized Caco-2 cell-monolayer shows localization of CSPP-L (a-CSPP-L, green) to desmosomal cell junctions (a-Desmoplakin, red).
(TIF)

S1 Video siGFP transfected Caco-2 spheroid. Animated optical sectioning through Z-stack of Caco-2 spheroid displayed in Fig 6D to emphasize lumen oriented localization of centrosomes. Stack is shown first with DNA (blue), centrosomes (Pericentrin, green) and filamentous actin (Phalloidin, red) and followed by a second optical sectioning omitting the actin signal.
(MP4)

S2 Video siCSPP1 transfected Caco-2 spheroid. Animated optical sectioning through Z-stack of Caco-2 spheroid displayed in Fig 6D to emphasize lumen oriented localization of centrosomes. Stack is shown first with DNA (blue), centrosomes (Pericentrin, green) and filamentous actin (Phalloidin, red) and followed by a second optical sectioning omitting the actin signal.
(MP4)

S3 Video siDesmoplakin transfected Caco-2 spheroid. Animated optical sectioning through Z-stack of Caco-2 spheroid displayed in Fig 6D to emphasize lumen oriented localization of centrosomes. Stack is shown first with DNA (blue), centrosomes (Pericentrin, green) and filamentous actin (Phalloidin, red) and followed by a second optical sectioning omitting the actin signal.
(MP4)
