# Supplementary figures and images for: CSPP-L Associates with the Desmosome of Polarized Epithelial Cells and Is Required for Normal Spheroid Formation
Source: PLoS One. 2015 Aug 4;10(8):e0134789. doi: 10.1371/journal.pone.0134789 (PMC4524657; doi:10.1371/journal.pone.0134789)

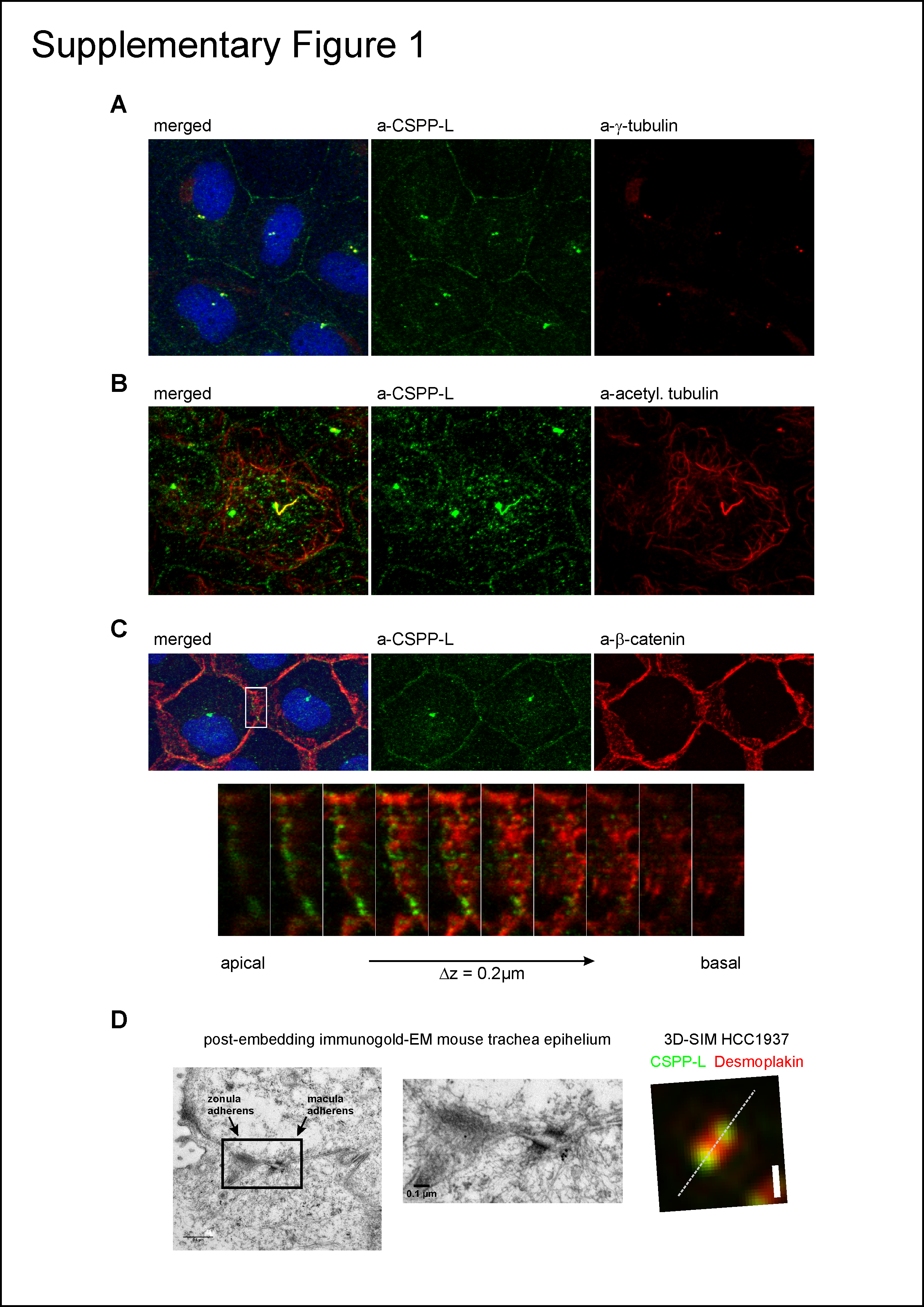

Supplement: S1 Fig — (A) IF of apical-basal polarized MDCK2 cell-monolayers showing localization of CSPP-L (a-CSPP-L, green) to (A) centrosomes (a-γ-tubulin, red), (B) primary cilia (a-acetylated tubulin, red), and (C) apical cell-cell junctions (a-β-catenin, red). (D) Post-embedding immunogold labeling of CSPP-L in mouse trachea epithelia cells shows CSPP-L staining in the vicinity of the desmosomal junction. A proportionally scaled 3D-SIM image of a HCC1937 cell Desmosome is shown for comparison. (TIF) [file pone.0134789.s001.tif]

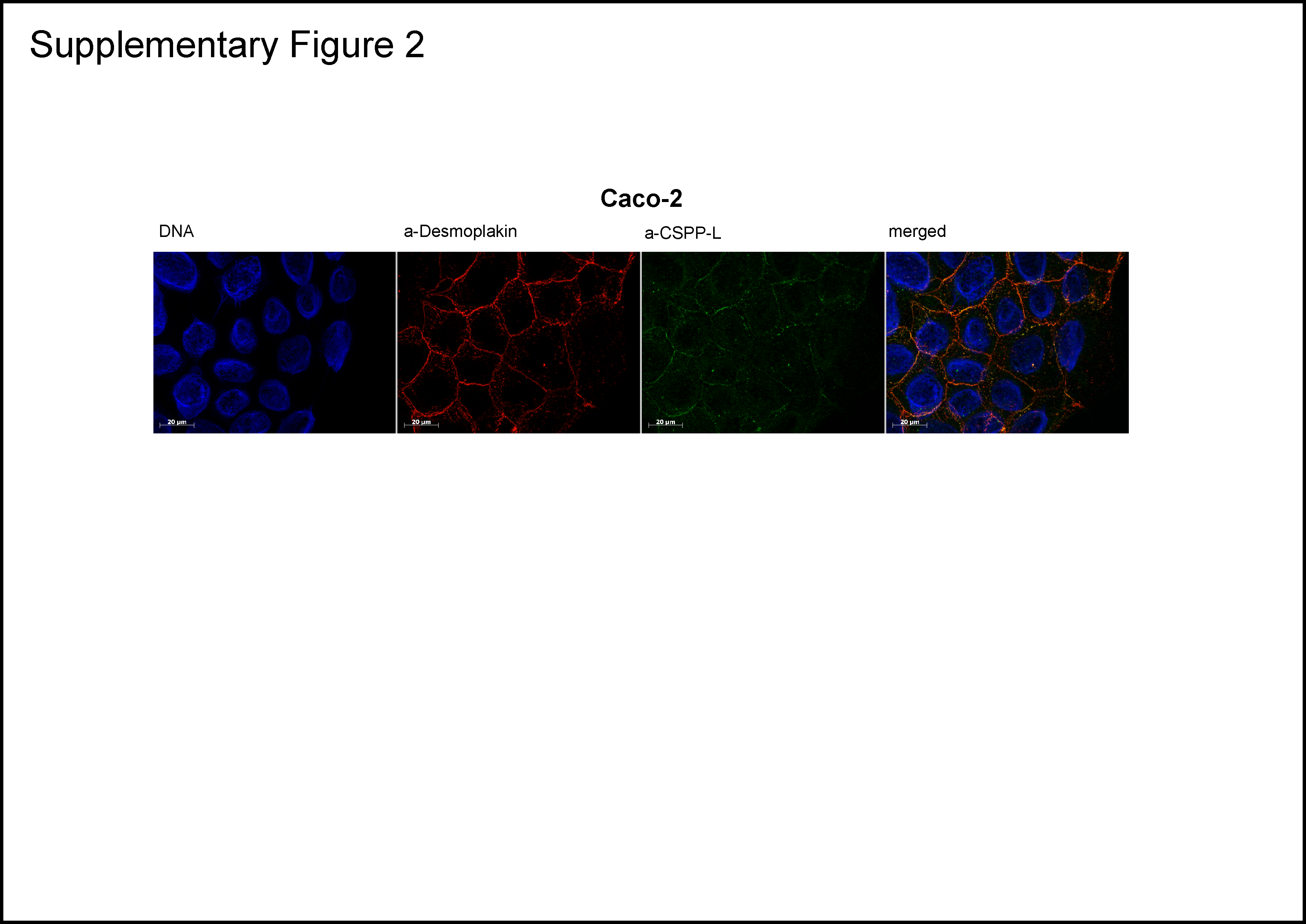

Supplement: S2 Fig — IF of apical-basal polarized Caco-2 cell-monolayer shows localization of CSPP-L (a-CSPP-L, green) to desmosomal cell junctions (a-Desmoplakin, red). (TIF) [file pone.0134789.s002.tif]
